# Optimizing the Work of Learning for Flourishing: A Randomized Pilot Study of a Digital CBT-Based Intervention in Medical Students

**DOI:** 10.1007/s40596-026-02308-w

**Published:** 2026-02-07

**Authors:** Justin S. Sanchez, Demetrius Perry, Anna Kinghorn, David Vermette, Teddi Gray, Jennifer G. Duncan, Brad Evanoff, Ginger E. Nicol

**Affiliations:** Washington University School of Medicine, St. Louis, MO USA

**Keywords:** Digital mental health, Cognitive behavioral therapy, Medical students, Burnout, Flourishing

## Abstract

**Objective:**

This study aimed to evaluate the feasibility, acceptability, and impact of a digital cognitive behavioral therapy (CBT)-based intervention to improve well-being in medical students.

**Methods:**

The authors conducted a 16-week randomized (1:1) controlled trial of a digital CBT-based intervention (OptimalWork) compared to a podcast listening control in medical students. Both interventions were fully remote, self-paced, and designed to take 10–15 min per day, 5 days per week for 4 weeks. Primary outcomes were feasibility and acceptability; measures of impact included flourishing (Flourish Index) and burnout (Professional Fulfillment Index) at 8 weeks. Qualitative analysis of open-ended feedback and exit interviews was performed to understand participants’ experience with the intervention, as well as barriers and facilitators of implementation.

**Results:**

Thirty-five medical students were enrolled and randomized. The OptimalWork group showed improvements in work engagement, burnout, anxiety, and stress, and an increase in overall flourishing. Dropout rates were high in both arms (46% by 4 weeks, 56% by 16 weeks), with lower baseline work engagement and higher stress predicting attrition. Qualitative analysis revealed that students who completed OptimalWork found it feasible, relevant, and helpful, while participants who dropped out cited time constraints and difficulty with daily habit formation.

**Conclusions:**

This pilot study demonstrates the feasibility of delivering a CBT-based digital intervention designed to improve well-being at work for medical students and offers insights into barriers and facilitators that can guide implementation efforts.

**Trial Registration:**

ClinicalTrials.gov identifier: NCT06914843 (July 24, 2024, retrospectively registered).

Medical students are more likely than the average person to experience mental illness and less likely to seek help [1]. Burnout—a syndrome consisting of emotional exhaustion, depersonalization, and a decreased sense of personal accomplishment [2]—has been estimated to affect between 33 and 55% of medical students [3], despite the fact that medical students begin training with lower rates of burnout and depression relative to the general population [4]. Far from being a benign condition, burnout has been shown to increase the risk of depression, anxiety, and suicidal ideation [5, 6], particularly in early-career physicians and trainees [7]. From an economic standpoint, burnout increases trainees’ likelihood of dropping out [8], and it is estimated that burnout in healthcare professionals costs the US healthcare system $4.6 billion per year related to turnover and reduced work hours [9].

Just as physical health is not merely the absence of disease [10], so the idea of “mental health” should represent more than simply the absence of burnout. The concept of “flourishing,” which includes psychological well-being as well as character, relationships, meaning, and purpose [11], may offer a positive reframe for the well-being problem in medical trainees [12]. Instruments designed to measure flourishing rather than burnout may be better suited to identify opportunities for intervention in trainees before they experience significant burnout, and initial work in this direction has been reported recently in medical residents [13] and medical students [14]. Moreover, interventions aimed at improving flourishing—including flourishing in work itself—may have advantages over conventional wellness efforts that risk reinforcing a work-life antagonism [15].

The causes of burnout include organization- and individual-level factors, and efforts to reduce burnout have addressed both sets of contributors [16]. Organizational factors, including workload demands, inefficiencies, and institutional culture, may be more difficult to modify, but interventions at this level can have a substantial impact [17]. At the same time, the observation that individuals vary in well-being even within systems suggests that individual-level factors (e.g., optimism, self-efficacy, resilience) may contribute to variable risk for burnout under similar workplace conditions [18]. A recent Cochrane Review of individual-level interventions for reducing occupational stress in healthcare workers found these interventions to be effective [19], although there have been relatively few studies of this type involving medical students [19, 20].

Given the efficacy of cognitive behavioral therapy (CBT) and mindfulness-based approaches for treating mood and anxiety disorders and reducing occupational stress [21], many individual-level interventions have focused on teaching people these psychological skills, including digital approaches that aim to overcome the barriers to the implementation of face-to-face therapy [22]. Although digital CBT-based interventions have been found feasible and effective in both preventing [23] and reducing existing distress [24] in controlled studies, implementation in real-world settings has been difficult [25], and successful interventions to improve mental health require a better understanding of the barriers and facilitators of implementing these interventions.

In this study, we performed a randomized pilot study of a digital intervention (OptimalWork) that teaches CBT- and mindfulness-based skills as they apply in daily life and work. The intervention was developed by a psychiatrist with students and trainees in mind, aiming to provide brief, structured content and exercises that can be integrated into one’s work. The focus of OptimalWork is learning to reframe everyday challenges in work and life as opportunities to grow according to ideals, integrate personal and professional identities, and deepen relationships with others. The skills taught are broadly applicable across contexts, but may be especially relevant for medical trainees who face challenges including heavy workloads, work-life conflicts, and loneliness [26], and whose initial idealism tends to erode during training [27].

Our primary aims were to assess the feasibility, acceptability, and preliminary effect size of this intervention in medical students. We recruited medical students irrespective of existing burnout or stress levels, with the goal of promoting flourishing rather than treating distress. We also sought to evaluate the OptimalWork Inventory as a measure of domains of well-being relevant to this population, and to identify barriers and facilitators to successful implementation through open-ended feedback and exit interviews.

## Methods

### Study Design and Participants

We conducted a 16-week randomized controlled trial, comparing the OptimalWork online program versus a Podcast Listening control group (described below). The intervention period lasted 4 weeks. Outcome measures (digital surveys) were sent via email at baseline, 4, 8, 12, and 16 weeks. The primary endpoint was 8 weeks, selected to assess the durability of any effects following the 4-week intervention, while immediate post-intervention effects (4 weeks) and longer-term outcomes (12 and 16 weeks) were assessed as secondary endpoints. The trial was registered on ClinicalTrials.gov (Identifier: NCT06914843). Enrollment began in September 2023; the trial record was created retrospectively in November 2023, but not formally released until July 2024. The study was approved as minimal risk by the Washington University School of Medicine Institutional Review Board and Medical Education Research Unit. Participants provided digital informed consent to participate using a self-guided digital consent framework.

Eligible participants were students currently enrolled at Washington University School of Medicine in St. Louis from all class years (M1–M4, including those on research years and MD/PhD students in the PhD years, total enrollment estimated at 600). All currently enrolled students were eligible regardless of baseline levels of burnout or distress. Presentations about the study were delivered to each class at the beginning of the academic year, and via IRB-approved recruitment emails sent by the school’s administrative office. Participants were reimbursed up to $50 in gift cards for participation ($10 each on completion of surveys at baseline and four follow-ups).

Participants were randomized 1:1 using a computer-generated allocation sequence prepared by an independent biostatistician and implemented using REDCap’s built-in randomization module [28]. After completion of the 8-week follow-up survey, participants were offered the option of switching to the other intervention, to ensure equity in access to the active intervention between groups.

### Interventions

#### OptimalWork

OptimalWork is a commercially available online program (OptimalWork.com), developed by a psychiatrist with expertise in CBT, Acceptance and Commitment Therapy (ACT), and mindfulness-based approaches, that teaches evidence-based psychological skills from these therapeutic approaches as they apply in one’s daily work and life. OptimalWork was chosen for this pilot because it can be delivered entirely remotely with a relatively low time burden, and its brief, structured content and exercises are intended to be integrated into one’s work, making it potentially well suited to the demands of medical education. OptimalWork has shown promise in promoting flourishing among its users, but this pilot represents its first controlled evaluation.

For the purposes of this study, participants were instructed to complete OptimalWork’s Daily Plan Recommendations, which consist of about 10–15 min of content per day, 5 days per week for 4 weeks. The Daily Plan Recommendations consist of one MasterClass lesson (5–7 min), one Audio Short (2–3 min), and one “Golden Hour” exercise (3–5 min). MasterClass Lessons include Tutorials (video content explaining the concept of the day) and Best Practices (interactive sessions that incorporate user input to provide practical suggestions on how to apply the concept in daily life). Audio Shorts serve to reinforce the topics from the MasterClass by applying them to common challenges. Finally, the “Golden Hour” tool guides the user to prepare one hour of their daily work as an opportunity to practice the principles from the MasterClass by applying the core skills of reframing, mindfulness, and behavioral activation to a task of the user’s choosing.

OptimalWork participants completed the OptimalWork Inventory (OWI) weekly through the application and received weekly, personalized advice on how to improve on one of its 24 items. The OWI, which has theoretical foundations in Acceptance and Commitment Therapy, was developed initially as a way for OptimalWork users to track progress. The OWI consists of 24 positively worded statements (e.g., “Instead of being overwhelmed, I get energized by challenges in my work and social life”), each rated on a scale of agreement from 1 to 10, with higher scores indicating better adaptive functioning. Items are structured around four theoretically derived subdomains: Ideals, Mode of Working, Work-Life Harmony, and Friendships.

The OWI demonstrated the following psychometric properties in an independent, cross-sectional sample of 835 OptimalWork users (university students and professionals): good convergent validity with the established Utrecht Work Engagement Scale (*r* = 0.647) [29], excellent internal consistency (Cronbach’s *α* = 0.921 overall), good subscale reliability (*α* = 0.70–0.89), and good subscale cohesion without excessive redundancy (*r̄* = 0.33–0.50). In this study, both groups completed the OWI at baseline, 4 weeks, and 8 weeks; we report descriptive statistics and baseline (i.e., pre-intervention) correlations with other measures of well-being.

#### Podcast Listening

Structured podcast listening served as an attentional control for this study, following a similar study of a web-based mindfulness intervention [30], because podcast listening would approximate the time and level of engagement required in the OptimalWork intervention and participation could be tracked online. Podcasts were taken from Radio Lab (RadioLab.org), a freely available podcast series that provides educational narratives on topics in biology, sociology, mathematics, cultural trends, and economics. A curated set of podcasts was specifically provided to exclude content related to CBT or mindfulness. For the purposes of this study, participants were instructed to listen to all 20 podcast clips (about 10 min each), adapted with permission from Basso et al. 2019 [30], with a suggested schedule of one per day, 5 days per week for 4 weeks.

#### Intervention Compliance Measures

Although the total amount of time spent per day on each intervention was designed to be similar, the structure of content differed between arms. Participants in the OptimalWork arm were invited to complete up to three activities per day (MasterClass lesson, Audio Short, and Golden Hour), for a suggested total of 60 engagements across 4 weeks. Engagement was tracked by objective app usage. The platform also includes a larger library of content (> 50 MasterClass lessons, > 80 Audio Shorts) that participants were free to explore. Participants in the Podcast arm were invited to complete 20 podcast modules over 4 weeks, each with a self-reported attestation of completion.

### Outcomes

#### Primary Outcomes

Our primary well-being outcomes at 8 weeks were the following: (1) Flourishing, assessed by the Flourish Index (FI) [11], a broad, well-validated measure covering five domains: Happiness and Life Satisfaction, Mental and Physical Health, Meaning and Purpose, Character and Virtue, and Close Social Relationships; and (2) Burnout, assessed by the Professional Fulfillment Index (PFI) burnout subscale [31].

The Flourish Index (FI) consists of 10 items rated 0–10, with higher scores indicating greater flourishing. The FI and its subdomains have demonstrated good reliability and construct validity across diverse populations, including healthcare workers and medical trainees [13]. We report the overall average FI score (0–10) and the five subdomain scores (0–10).

The Professional Fulfillment Index (PFI) Burnout scale consists of two domains: Interpersonal Disengagement (6 items) and Work Exhaustion (4 items). This scale has shown good internal consistency (*α* = 0.92), high correlation with the Maslach Burnout Inventory (*r* > 0.5), and sensitivity to detect burnout in healthcare workers including residents and fellows [31]. Items are rated on a 5-point Likert scale from “Never” to “Very Often,” and conventionally reported as a numeric average between 0 and 4, with scores > 3 indicating clinically significant symptoms [31]. We report the total burnout score (mean of all 10 items) as well as the subdomain scores.

#### Secondary Outcomes

Four additional psychological domains were assessed as secondary outcomes: Emotional Support, Anxiety, Depression, and Stress. These domains were chosen based on their relevance to medical trainees and the availability of brief, well-validated instruments to minimize participant burden, described below.

Emotional Support, Anxiety, and Depression were measured with short-form scales from the Patient-Reported Outcomes Measurement Information System (PROMIS), an NIH initiative to create standardized, reliable, and valid patient-reported measures across multiple domains of health and well-being [32]. For this study, we administered (1) the 4-item Emotional Support scale, (2) the 4-item Anxiety scale, and (3) the 4-item Depression scale. These short forms were selected to minimize participant burden while retaining adequate coverage of each construct. Responses are recorded on a 5-point Likert Scale (Never–Always), with higher scores indicating more of the construct measured over the past seven days. We analyzed the average item score (range 1–5) for each measure.

Stress was assessed using the Brief Inventory of Perceived Stress (BIPS) [33], which was chosen to minimize participant burden while capturing domains of stress relevant to medical trainees. The BIPS contains 9 items across three subdomains: Pushed, Conflict and Imposition, and Lack of Control. Items are rated on a 5-point Likert scale, with higher scores indicating greater perceived stress. The BIPS has been validated in samples including first-year health science graduate students [34]. Following convention, we report the total score as a sum (range 0–36), and the three subdomains as average item scores (range 1–5).

#### Demographics and Expectancy

In addition to well-being measures described above, participants completed a baseline survey assessing (i) demographics, (ii) work context/environment (e.g., classroom, clinical, research), (iii) credibility/expectancy related to the intervention, and (iv) familiarity and prior experience with CBT. Demographics included age, gender, race, ethnicity (Hispanic/Latino), entering class year, and Medical Scientist Training Program (MSTP, i.e., MD/PhD) status. For credibility/expectancy, participants were asked how logical the intervention seemed to them and how helpful they expected it would be [35]. Participants were also asked about prior familiarity with CBT.

### Qualitative Data

At the end of the 4-week intervention, participants from both groups were asked to respond to two open-ended survey questions: (1) “Please comment on the feasibility of completing the program as a medical student,” and (2) “Please comment on what worked well and what could be improved with the program.” We also planned to conduct exit interviews with a subset of participants who either enrolled but did not begin the intervention, or who began but did not complete the intervention. Email invitations to participate in exit interviews were sent to all participants who dropped out at any point before week 4; however, only two responses were obtained.

#### Qualitative Data Analysis

We used a thematic analysis approach [36] to analyze open-ended survey responses. Our goal was to identify a few common themes, barriers, and facilitators, although we could not sample to saturation or sufficiency of themes due to sample size. First, all responses and transcripts were read multiple times to achieve familiarity with the data. Subsequently, codebooks were developed inductively by two coders collaboratively to capture recurrent concepts or issues raised by participants. Codes were then applied to the datasets independently by two coders, and discrepancies were discussed until consensus was reached. The final codes were tabulated and representative comments were selected to illustrate key findings.

### Statistical Analyses

Analyses of well-being outcome measures were conducted to estimate effect sizes and characterize trajectories to inform future confirmatory trials. Changes in well-being measurements at 8 weeks—Flourishing (FI) and Burnout (PFI)—served as the primary well-being outcomes. We used linear mixed-effects models to analyze trajectories in well-being measures and intervention effects. Each model included time (in weeks), study arm, and their interaction as fixed effects, with a random intercept for participant to account for repeated measures. Models were fit separately for each outcome variable and for follow-up horizons of 4 and 8 weeks. Participants with missing follow-up data were retained in the models, contributing data from earlier time points when available. As this was a pilot study, these analyses primarily aimed to estimate effect sizes to guide future confirmatory trials, rather than to provide definitive hypothesis tests.

Exploratory analyses included between-group comparisons of trajectories of subdomains of the FI and PFI, as well as secondary outcomes (Emotional Support, Anxiety, Depression, Stress), modeled as above. Baseline group differences in demographics, baseline expectations (logic/helpfulness), and feasibility/acceptability ratings were assessed with 2-sample *t*-tests for continuous variables or chi-squared tests for categorical variables. Within-group pre-post changes in well-being measures and expectancy questions were assessed using paired *t*-tests.

Post hoc analyses were conducted to explore relationships between demographic variables and well-being measures, using *t*-tests for variables with 2 groups or ANOVA for variables with > 2 groups. Cross-sectional (baseline) relationships between OWI and other well-being measures and their subdomains were assessed with Pearson correlations. Baseline predictors of study retention were assessed in a similar manner comparing completers and dropouts. Statistical analyses were performed using R (v. 4.2.2).

## Results

### Participant Characteristics

**Fig. 1:**
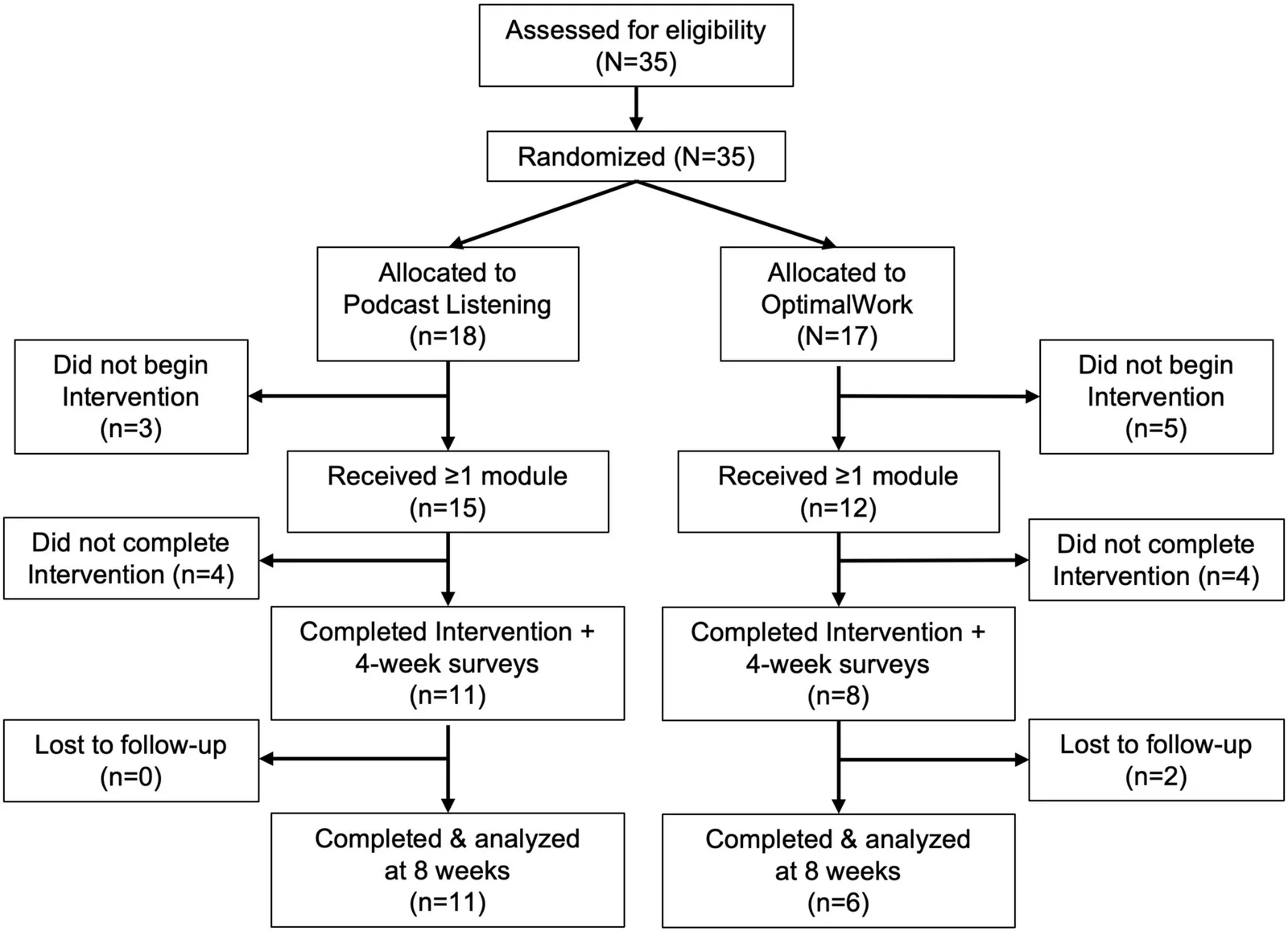
CONSORT flow diagram. Of 35 participants assessed for eligibility, none was excluded. All 35 were randomized 1:1 to Podcast Listening (*n* = 18) or OptimalWork (*n* = 17). Primary analyses were conducted by intention-to-treat, including all randomized participants

Baseline characteristics of enrolled participants are shown in Table 1. A total of 35 students were enrolled and randomized: 17 to the OptimalWork group and 18 to the Podcast group. Participants represented all four years of medical school (M1–M4), including five students completing a research year for a master’s or PhD program. The two groups did not differ significantly in baseline demographics. Participant flow and study retention are summarized in Fig. 1.

**Table 1:** Participant characteristics. Continuous variables displayed as mean ± standard deviation [min–max], categorical variables as *N* (%) or *N* in each group. *p*-values correspond to 2-sample *t*-tests for continuous variables or chi-squared tests for categorical variables

	OptimalWork	Podcast	All	*p*-value
***N***	17	18	35	—
**Age** *Years*	25.4 ± 2.5 [22.0–31.0]	25.1 ± 2.3 [22.0–30.0]	25.2 ± 2.4 [22.0–31.0]	0.721
**Gender** *Male, N (%)*	6 (35.3)	4 (22.2)	10 (28.6)	0.630
**Race** *Asian | White | Other*	5 | 9 | 3	7 | 8 | 3	12 | 17 | 6	0.834
**Year in School** *M1 | M2 | M3 | M4/Other*	4 | 3 | 7 | 3	6 | 3 | 5 | 4	10 | 6 | 12 | 7	0.838
**MD/PhD Program** *N (%)*	4 (23.5)	4 (22.2)	8 (22.9)	1.000
**Work Context** *Preclinical | Clinical | Other*	7 | 7 | 3	9 | 6 | 3	16 | 13 | 6	0.861
**Work Engagement (OWI)** *Scale of 1–10*	5.3 ± 0.9 [4.2–7.1]	5.8 ± 1.2 [4.0–7.8]	5.6 ± 1.1 [4.0–7.8]	0.163
**Flourishing (FI)** *1–10*	6.8 ± 1.3 [2.5–8.5]	6.9 ± 1.3 [3.8–8.8]	6.9 ± 1.3 [2.5–8.8]	0.879
**Burnout (PFI)** *0–4*	1.4 ± 0.7 [0.1–2.4]	1.4 ± 0.6 [0.4–2.3]	1.4 ± 0.7 [0.1–2.4]	0.903
**Expectancy—How logical?** *1–5*	3.1 ± 0.7 [2.0–4.0]	3.6 ± 0.8 [2.0–5.0]	3.3 ± 0.8 [2.0–5.0]	0.050
**Expectancy—How helpful?** *1–5*	2.9 ± 0.7 [2.0–4.0]	3.0 ± 0.7 [2.0–4.0]	2.9 ± 0.7 [2.0–4.0]	0.618
**CBT Familiarity** *1–5*	3.5 ± 1.1 [1.0–5.0]	3.6 ± 0.9 [2.0–5.0]	3.6 ± 1.0 [1.0–5.0]	0.816

In response to questions about intervention expectations at baseline, the Podcast group rated their intervention as seeming more logical (mean 3.6/5 vs. 3.1/5, *p* = 0.05), while both groups gave similar ratings for anticipated helpfulness (mean 3.0/5 vs. 2.9/5, *p* = 0.618). Self-rated familiarity with cognitive behavioral therapy (CBT) did not differ significantly between groups (mean 3.5/5 vs. 3.6/5, *p* = 0.816).

After 8 weeks, two participants from the Podcast group opted to try the OptimalWork intervention, while none from the OptimalWork group chose to access the Podcast program.

### Feasibility and Acceptability

#### Quantitative Data

Participant responses to questions about four feasibility and acceptability domains are summarized in Table 2. After completion of the intervention period, participants in both groups reported high satisfaction with the content organization and user-friendliness of the interventions. Ratings for the amount of time spent and feasibility of completing in the allotted time were also favorable (> 3.5/5) in both groups. Group differences between study arms in feasibility and acceptability measures were not statistically significant (*p* > 0.2).

**Table 2:** Post-intervention feasibility and acceptability data. Descriptive statistics (mean, standard deviation, % of responses ≥ 4/5) are shown for Likert scale responses (range 1–5) to questions about feasibility and acceptability of the interventions, assessed immediately after completion of the intervention period (end of week 4). Two expectancy items (how logical/how helpful does the program seem to you?) were assessed before and after the intervention (bottom half of table)

	OptimalWork (*n* = 8)		Podcast (*n* = 11)	
1 = Very unsatisfied, 5 = Very satisfied	Mean (SD)	% ≥ 4	Mean (SD)	% ≥ 4
Organization of content	4.38 (0.52)	100.0	4.27 (0.47)	100.0
User-friendliness of the application	4.38 (0.52)	100.0	4.00 (1.18)	81.8
Amount of time spent	4.13 (1.13)	75.0	4.36 (0.67)	90.9
Feasibility of completing in allotted time	3.88 (1.13)	62.5	4.36 (0.81)	81.8
1 = Not at all, 5 = Very				
**Perceived logic of the program**				
Pre (baseline)	2.88 (0.64)	12.5	3.55 (0.93)	45.5
Post (week 4)	3.88 (0.83)	62.5	3.09 (1.22)	36.4
**Perceived helpfulness of the program**				
Pre (baseline)	2.88 (0.83)	25.0	2.91 (0.70)	18.2
Post (week 4)	3.50 (1.31)	62.5	2.73 (1.56)	36.4

We also assessed participants’ perception of the logic and helpfulness of the interventions, both before and after the intervention period. Participants in the OptimalWork group reported increases in both perceived logic (*M* = 2.88 to 3.88, paired *t*-test *p* = 0.03) and helpfulness (*M* = 2.88 to 3.50, *p* = 0.28) of the program, whereas ratings in the Podcast group remained stable or declined slightly over time (Table 2). Between-group differences in these ratings were not statistically significant (*p* > 0.2).

Intervention compliance also differed by study arm. In the Podcast group, among participants who completed the 4-week follow-up surveys, participants completed a mean of 17.7 modules (median 20; range 1–20) out of the 20 assigned. In the OptimalWork group, participants completed a mean of 24 total engagements (median 16, range 3–80) out of a suggested 60 possible. Of the three content types on OptimalWork, participants completed the highest number of MasterClass lessons (mean 10.7), followed by Audio Shorts (8.1) and Golden Hours (5.6).

#### Qualitative Data

Qualitative analysis of responses to open-ended feedback prompts is summarized in Table 3. Responders in both groups generally reported that the interventions were feasible to complete. Several participants noted that the daily time commitment was manageable, e.g., “I felt that the short daily modules were very easy to get done quickly each day.”

**Table 3:** Themes identified in qualitative feedback. Thematic analysis of open-ended post-intervention survey data revealed themes shown in rows. Themes were induced from the data by iterative coding and discussion among two coders, and fell into two broad categories: facilitators and barriers. One additional theme, “platform recommendations,” was also identified. Representative comments from either group are shown, -- indicates there were no comments fitting that theme for that group

		OptimalWork arm (*N* = 8)	Podcast arm (*N* = 8)
	Theme	Representative comment	Representative comment
**Facilitators**	Content applicable	“there are plenty of opportunities during the day of a medical student where the techniques learned can be applied”	--
	Content helpful	“I normally do feel more ready for the day following the activities”	--
	Content interesting	“I also enjoy that the courses included more activities throughout (breathing exercises or questions)”	“I thought the content of the Podcasts were interesting”
	Golden Hour helpful	“I particularly loved the golden hour tool and used it frequently outside of the daily tasks”	--
	Content amount feasible	“It only took <10 min which is easy to fit into a daily schedule”	“It was very feasible to complete the program”
	Content structure helpful	“The Master Class did an excellent job of breaking up the idea of flourishing into smaller achievable steps"	“I think the length of the clips was good”
	Platform easy to use	“Easy to access with intuitive software”	“I felt the Canvas page was very easy to navigate”
**Barriers**	Content unhelpful	“Honestly, I don’t think it helped”	“I wasn’t a very big fan of the podcast content (didn’t find it very helpful or motivating)”
	Content uninteresting	--	”I paid less and less attention to them and found myself not interested”
	Platform issues	“There were times I did my masterclass late … and then the next day there were not new lessons”	“There were sometimes glitches … where it would seem not to register my attestation”
	Content amount challenging	”I struggled in keeping myself accountable to using it on a weekly basis”	“On very busy weeks, it was a little more difficult to listen to the podcast”
	Content structure unhelpful	--	“I would have liked to listen to a whole episode at a time not just a part”
**Other**	Platform recommendations	“A calendar invite might be useful to help remember”	“daily pop-up reminders would have made it easier to complete everything in a timely manner”

In terms of acceptability, participants in the OptimalWork group (*n* = 8) described the content as helpful, interesting, and relevant to their daily experiences as medical students (e.g., “It gave me a sense of calmness and allowed me to take charge of the day”). Several participants referenced OptimalWork’s “Golden Hour” tool as particularly helpful. One OptimalWork participant commented that they thought the program did not help them. In the Podcast group (*n* = 8), several participants described the content as interesting, none described it as helpful or applicable, and some reported difficulty staying engaged due to lack of interest.

Barriers most often cited were related to time pressures and difficulty sustaining regular use, even when the content was well-received. This challenge was noted in both groups, e.g., “I struggled in keeping myself accountable to using it on a weekly basis.” Participants across both groups also shared suggestions for improvement, such as adding daily reminders to support engagement.

### Study Retention, Facilitators, and Barriers

We next examined retention over time and factors associated with dropout. Nineteen of 35 participants (54%) completed the study through the 4-week intervention period (i.e., completed the 4-week follow-up surveys), and 16 (46%) completed through the 16-week follow-up. Most attrition occurred either prior to intervention activation or during the intervention period (Fig. 1). Dropout rates did not differ significantly between the OptimalWork and Podcast groups during the 4-week intervention period (*p* = 0.625) or over the full study (*p* = 0.129).

To identify potential predictors of retention, we compared baseline demographic and well-being measures between participants who dropped out versus those who completed the study. No significant differences were observed based on age, gender, year in school, MD/PhD status, or expectations about the interventions. However, participants who dropped out in the first 4 weeks reported higher baseline levels of perceived stress (BIPS Conflict subdomain, *p* = 0.074; Feeling Pushed subdomain, *p* = 0.093) compared to those who completed the intervention period. Additionally, participants who dropped out had significantly lower baseline scores on the OptimalWork Inventory compared to those who finished through 16 weeks (*M* 5.2 vs. 5.8, *p* = 0.02), suggesting that baseline well-being and adaptive functioning may have affected participants’ ability to implement these programs.

### Well-being Measures

Baseline values for well-being measures are shown in Table 1; these measures did not differ significantly between the OptimalWork and Podcast groups at baseline.

**Fig. 2:**
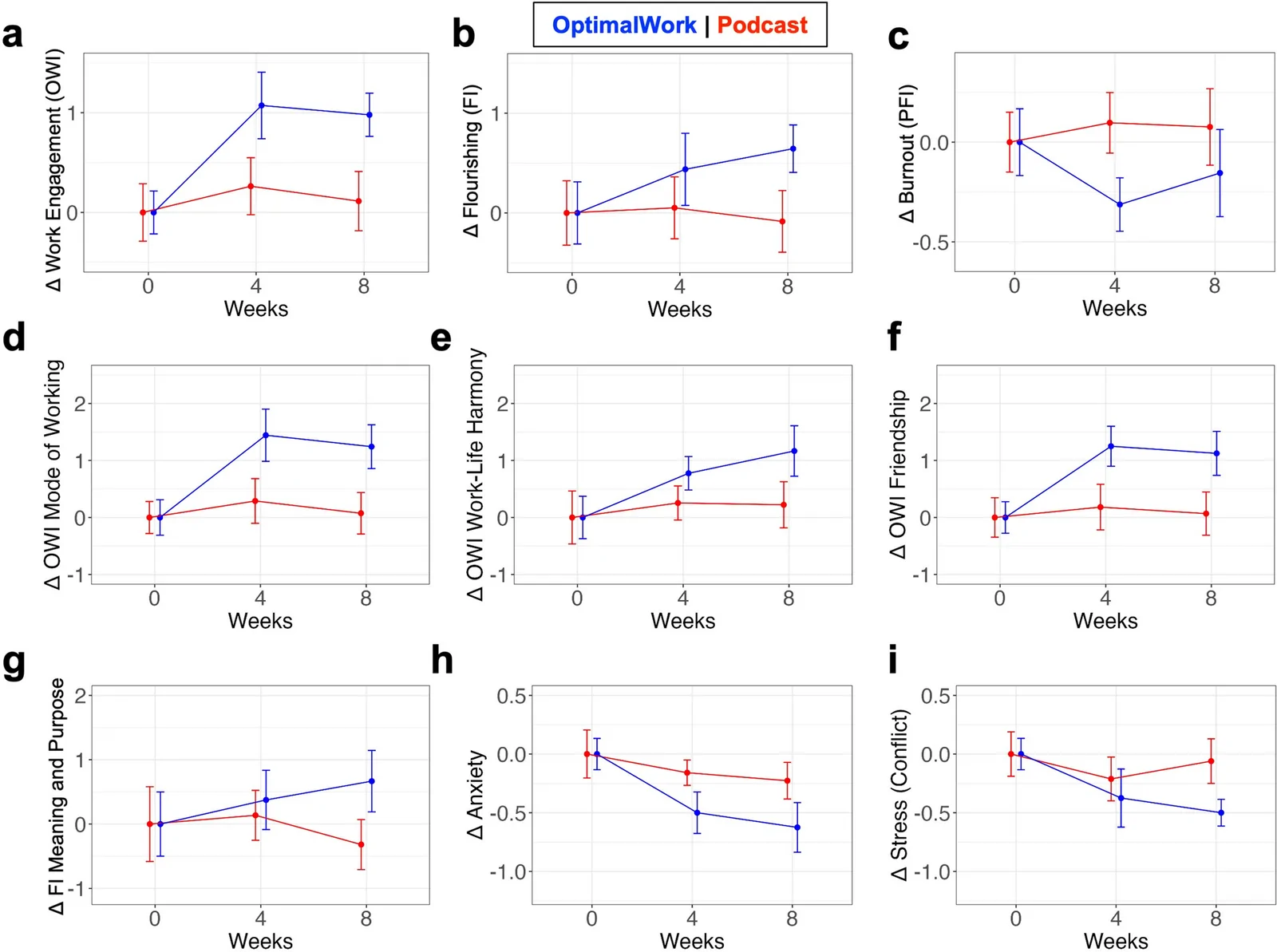
Change in well-being measures by group. Change from baseline (Δ) in **a** Work Engagement, assessed by the OptimalWork Inventory (OWI), **b** Flourishing, assessed by the Flourish Index (FI), and **c** Burnout, assessed by the Professional Fulfillment Index (PFI) Burnout subscale. OptimalWork Inventory (OWI) subdomains: **d** Mode of Working, **e** Work-Life Harmony, **f** Friendships. **g** Flourish Index subdomain of Meaning and Purpose; **h** Anxiety as measured by PROMIS 4-item scale; **i** Stress as measured by the Brief Inventory of Perceived Stress, Conflict subscale. Higher scores are favorable for most measures, except Burnout (**c**), Anxiety (**h**), and Stress (**i**). Error bars represent mean ± standard error of the mean

Changes in well-being measures over time by group are shown in Fig. 2. OptimalWork participants increased significantly in FI total scores (out of 10) from baseline to 8 weeks (*M* ± SD change = 0.64 ± 0.58, 9.2% increase from baseline, paired *t*-test *p* = 0.042), although the group comparison model was not significant (*β* ± SE = 0.091 ± 0.057, *p* = 0.118). Within the FI subdomains, the largest effect was seen in the domain of Meaning and Purpose (0.124 ± 0.069, *p* = 0.081) (Fig. 2g). A significant reduction in Burnout (PFI) scores (out of 5) in the OptimalWork group was observed at 4 weeks (*M* ± SD change = −0.42 ± 0.36, *p* = 0.036) but not 8 weeks (*p* = 0.51), and between-group comparison models were not significant (4 weeks: *β* ± SE = −0.105 ± 0.052, *p* = 0.058; 8 weeks: −0.031 ± 0.032, *p* = 0.347).

Reductions in perceived stress (BIPSS – Conflict subscale) and anxiety (PROMIS 4-item) (Fig. 2h, i) over 8 weeks were observed in the OptimalWork group (*M* ± SD change = −0.50 ± 0.28, anxiety: −0.62 ± 0.52), although between-group differences were not statistically significant (stress: *β* ± SE = −0.051 ± 0.036, *p* = 0.171, anxiety: −0.043 ± 0.028, *p* = 0.135). Post hoc analyses examining relationships between demographic factors (e.g., year in training, work environment) and baseline well-being measures did not reveal statistically significant findings or consistent patterns.

### OptimalWork Inventory

At baseline, OWI total scores were positively correlated with the FI (*r* = 0.50, *p* = 0.002) and negatively correlated with the burnout (PFI) score (*r* = −0.43, *p* = 0.008), and FI and PFI were negatively correlated (*r* = −0.60, *p* = 0.0001). The average (*M* ± SD) improvement in OWI scores (out of 10) was 1.0 ± 0.5 (17.6% increase from baseline) for the OptimalWork group and 0.1 ± 1.0 (2.9% increase) for the Podcast group (Fig. 2a). Among OWI subdomains, the largest increases for the OptimalWork group were seen in Mode of Working (*M* ± SD = 1.24 ± 0.94), Work-Life Harmony (1.17 ± 1.08), and Friendships (1.12 ± 0.95) (Fig. 2d–-f).

## Discussion

In this 16-week randomized pilot study, we demonstrated the feasibility, acceptability, and preliminary effect size estimates of a 4-week digital CBT-based intervention (OptimalWork) for improving well-being in medical students. Despite the small sample size, quantitative trends in several well-being domains including flourishing, burnout, anxiety, and stress were found to favor the OptimalWork group. Participants generally found the interventions feasible and acceptable based on both quantitative and qualitative post-intervention survey data, and we identified barriers and facilitators from qualitative analysis. Overall, our findings suggest the promise of CBT-based digital interventions to promote flourishing in medical students and offer insights that can guide future implementation efforts.

Although users who completed the interventions rated them to be feasible and acceptable, the dropout rate in our study was high—46% dropped out by 4 weeks and 56% by 16 weeks. However, the dropout rate we observed was not dissimilar from other interventions of this kind. For example, another trial of a digital CBT-based program in graduate health science students had a dropout rate of 53% at 12 weeks [24], while a low-intensity internet intervention in medical professionals had a dropout rate of 82.5% at 6 weeks [37]. Some of this dropout may have been mitigated if we had included a run-in period prior to randomization to increase post-randomization retention. These kinds of study design considerations are of particular importance for improving retention in fully remote trials.

Qualitative feedback from participants highlighted several barriers to engagement, including time constraints and difficulty forming and maintaining a daily habit. These findings are consistent with prior work identifying barriers medical students face in implementing digital mental health interventions [38], and suggest strategies to improve retention in future work such as decreasing the amount of required content, increasing the number of reminders, or simplifying the content structure. Indeed, although the estimated time per day was designed to be similar between arms, OptimalWork divided this time into three separate engagements, whereas the Podcast arm offered a single daily module. Completion relative to suggested totals was higher in the Podcast arm, which may partly reflect the simplicity of its structure, notwithstanding differences in how compliance was measured (self-report vs. app usage). Within OptimalWork, participants engaged most with the MasterClass lessons (presented first each day), and least with the Golden Hour exercises (presented last). Future trials may benefit from simplifying the intervention structure or varying the order or presentation of content.

Quantitative analysis of factors predicting study dropout revealed that participants who dropped out had higher baseline levels of perceived stress and lower baseline work engagement scores. These findings suggest the existence of virtuous and vicious cycles, as has been noted in previous work on digital mental health interventions [25]: individuals with better adaptive functioning may be more likely to engage with and benefit from the intervention, whereas those who could benefit most from the intervention may be the least likely to complete it. Given the increased risk of burnout in early-career physicians [39], there is a strong case to be made that earlier intervention in the medical training pipeline (e.g., undergraduate pre-medical curricula), when medical trainees may have fewer obligations and lower perceived stress, could lead to better retention and outcomes [7].

The OptimalWork Inventory (OWI) was developed alongside the OptimalWork intervention to assess attitudes and behaviors targeted by the program. While we did not use it as an independent outcome measure in this pilot study, we found that this measure was correlated cross-sectionally with the FI and the PFI, suggesting convergence with these well-validated measures of well-being and distress, while also capturing variance not explained by these other measures. The quantitative improvement in OWI observed in OW users triangulates well with qualitative feedback from users that they found the intervention applicable and helpful in approaching challenges in their everyday work and life. Together, these findings suggest that the OptimalWork Inventory shows promise as a measure of domains of well-being relevant to medical students, and may be useful for medical educators or as an independent outcome measure in future studies.

Considering implementation and scalability of this intervention, it is noteworthy that OptimalWork is delivered entirely online and could be implemented with relatively low staffing burden. Implementation in a medical school setting would primarily require institutional endorsement and integration with existing student wellness resources, which could be facilitated by stakeholders such as student affairs or wellness offices. Regarding cost, premium subscriptions to the OptimalWork platform were provided for the purposes of this study; subscription models may evolve over time and exact subscription costs would be negotiated on an institutional basis. As the costs of development, software maintenance, and technical support are already absorbed by the company, anticipated institutional costs would primarily consist of subscription fees and any promotion or administrative support the school elects to provide. Published evaluations of similar digital mental health interventions suggest that the cost of delivering these interventions is lower than the cost of developing and maintaining them, and the per-user cost decreases with increasing scale [40].

Strengths of this study include the randomized design with use of an active control group, wide range of quantitative outcome measures, and qualitative feedback to identify barriers and facilitators. At the same time, several important limitations warrant consideration. The small sample size (*N* = 35) limits statistical power and the precision of effect estimates. As a pilot study, we conducted multiple exploratory analyses, and these results should be interpreted cautiously and not viewed as definitive hypothesis tests. High attrition further limits interpretation and may have introduced bias, as dropouts may have differed systematically from completers in ways we could not assess. Our quantitative and qualitative analyses of barriers and facilitators were similarly underpowered, limiting our ability to draw conclusions about participants’ experiences. Finally, this study was conducted at a single institution, and voluntary participation may have introduced self-selection bias, limiting generalizability to other medical student populations.

In summary, our findings highlight the promise of CBT-based digital interventions to promote flourishing in medical students and provide a foundation for future efforts to evaluate the effectiveness and implementation of these interventions. Future directions include scaling to larger, multi-site trials with longer follow-up, refining outcome selection, and integrating strategies to reduce participant burden and improve retention.

## Data Availability

Data supporting the findings of this study, as well as survey materials and additional analyses, are available from the corresponding author upon reasonable request.
